# Unique B- and plasma cell signature differentiates hidradenitis suppurativa from psoriasis and atopic dermatitis

**DOI:** 10.3389/fimmu.2026.1768249

**Published:** 2026-04-16

**Authors:** Conor M. Smith, Roisin Hambly, Solene Gatault, Luis F. Iglesias-Martinez, Sean Kearns, Helen Rea, Kate Lynam-Loane, Shivashini Kirthi, Rosalind Hughes, Walter Kolch, Jean M. Fletcher, Brian Kirby

**Affiliations:** 1 School of Biochemistry and Immunology, Trinity Biomedical Science Institute, Trinity College Dublin, Dublin, Ireland; 2 Charles Institute of Dermatology, School of Medicine, University College Dublin, Dublin, Ireland; 3 The Charles Centre, Department of Dermatology, St Vincent’s University Hospital, Dublin, Ireland; 4 School of Medicine and Medical Sciences, University College Dublin, Dublin, Ireland; 5 Systems Biology Ireland, School of Medicine, University College Dublin, Dublin, Ireland; 6 Clinical Research Centre, St Vincent’s University Hospital, Dublin, Ireland; 7 Conway Institute of Biomolecular and Biomedical Research, University College Dublin, Dublin, Ireland; 8 School of Medicine, Trinity Biomedical Science Institute, Trinity College Dublin, Dublin, Ireland

**Keywords:** atopic dermatitis, hidradenitis suppurativa, psoriasis, RNA-Seq - RNA sequencing, transcriptomic analysis

## Abstract

**Introduction:**

Hidradenitis suppurativa (HS), psoriasis (PsO), and atopic dermatitis (AD) have unique disease pathogenesis and distinct pathology, despite sharing some immunological pathways. This study aimed to evaluate the transcriptomic differences between HS, PsO, and AD lesions, which drive these unique pathogenic features.

**Methods:**

Bulk RNA sequencing was performed on 19 healthy controls (HC), 15 HS, 21 PsO, and 15 AD lesional skin samples.

**Results:**

PsO lesions were defined by a strong interleukin (IL)-17 signature, and AD lesions had increased T helper (Th2) signaling. HS lesions had a more heterogeneous inflammatory profile involving TNF, IL-17, B cells, and NLR signaling. Weighted gene coexpression network analysis identified T-cell signaling and epidermal development as important characteristics of PsO lesions, while HS lesions were defined by complement activation, B-cell signaling, and fibroblast-associated pathways consistent with potential scarring in HS. Finally, gene expression analysis identified increased expression of B-cell-, plasma cell-, and immunoglobulin-associated genes in HS lesions relative to HC skin, PsO, and AD lesions.

**Discussion:**

These data suggest that HS pathogenesis is more heterogeneous than AD or PsO and highlight an important role of B and plasma cells in HS inflammation.

## Introduction

The recurrent chronic inflammatory skin disease hidradenitis suppurativa (HS) affects ~ 1% of the population and is characterized by painful inflammatory nodules and the distinct development of deep-seated sinus tracts. Despite its distinct pathological features, HS shares immunopathology with psoriasis (PsO) and atopic dermatitis (AD). Th17 cells are central to PsO pathogenesis and are also implicated in HS (1–3). Previous RNA-sequencing (RNA-seq) studies have highlighted AD as a Th2-mediated disease, with increased expression of interleukin (IL)-13 correlating with disease activity (4). Despite being primarily driven by Th2 cells, there is a clear association between HS and AD, with AD being twice as common in HS patients compared with the general population (5, 6).

Immune dysregulation drives HS inflammation, with elevated tumor necrosis factor (TNF)-α reported in HS. TNF subsequently became the first biological target licensed for HS treatment (7, 8). More recently, Th17 cells and the associated cytokines IL-17, IL-1b, and IL-23 have been found to be elevated in HS skin (3, 9, 10). Patients with PsO benefit from IL-17-targeted therapies; however, only ~ 46% of patients with HS respond to secukinumab (11–13). Dual blockade of both IL-17A and IL-17F with bimekizumab is more effective in PsO than IL-17A inhibition alone with secukinumab (14); however, a similar head-to-head trial has yet to be performed in HS (12, 13, 15). IL-17 receptor inhibition with brodalumab is effective in HS, albeit in a limited number of patients in open-labeled trials (16, 17). Targeting upstream of Th17 cells with IL-23 inhibitors has yielded contrasting results in HS and PsO. In PsO, guselkumab has proven more effective than IL-17A inhibition with secukinumab; guselkumab only benefitted a subgroup of patients with HS (18) (NOVA trial, NCT03628924).

These contrasting responses to treatment in HS and PsO suggest that a better understanding of HS pathogenesis is required to develop effective therapeutic strategies. While comparisons between PsO and HS have been made (19, 20), to date, these diseases have not been directly compared with AD. In this study, we show that a unique B- and plasma cell signature exists in HS that is absent in other inflammatory skin diseases, suggesting a crucial role of B and plasma cells in HS pathogenesis.

## Materials and methods

**Figure 1:**
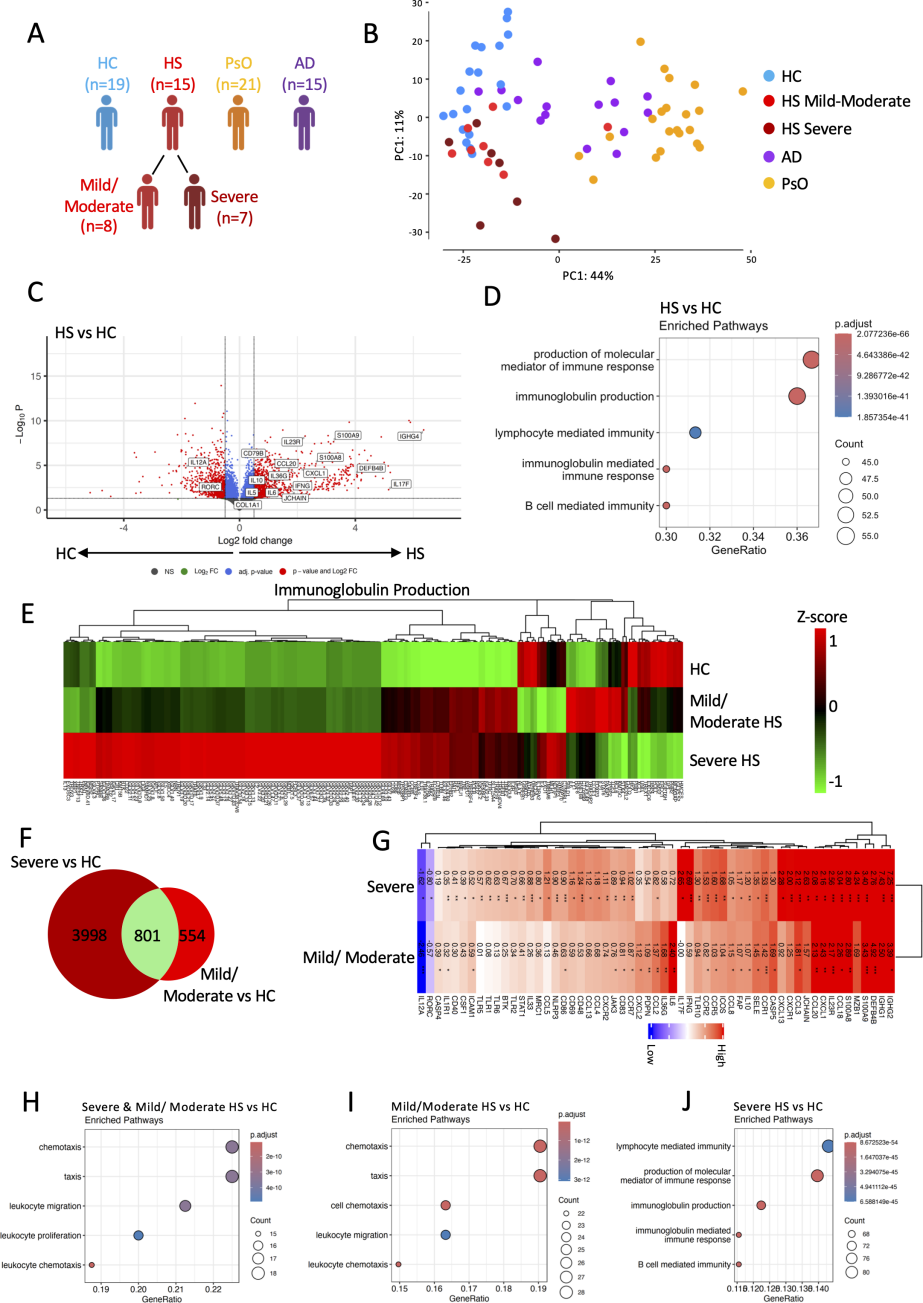
Bulk RNA sequencing identi ed differences between hidradenitis suppurativa (HS) lesional skin and healthy control (HC) skin **(A)**. Principal component analysis highlights the transcriptomic differences between HS (IHS4 mild–moderate, red, n = 8; IHS4 severe, dark red, n = 7), PsO (orange, n = 21), AD (purple, n = 15), and healthy control skin (blue; n = 19) **(B)**. Differentially expressed genes between HS and HC are visualized by a volcano plot. Genes with elevated expression in HS lesions have a negative log2 fold change, whereas genes with higher expression in HC have a positive fold change. The–log10 p-value on the y-axis represents the measure of signi cance **(C)**. Top ve gene ontology terms that are signi cantly enriched (p-value < 0.01 and q-value < 0.01 following BH adjustment) in the differentially expressed genes (both up- and downregulated) in HS lesional skin **(D)** are shown. Heatmap displaying the relative expression of genes involved in the GO:0002377 immunoglobulin production pathway **(E)**. Number of genes signi cantly up- and downregulated in mild/moderate and severe HS relative to HC **(F)**. Heatmap displaying the log fold change of genes associated with in ammation in mild/moderate and severe HS relative to HC **(G)**. Top ve gene ontology terms enriched from the differentially expressed genes unique to mild/moderate **(I)** and severe HS **(J)**, or shared **(H)** between them, when compared with HC skin.

Patients with HS were recruited as part of an investigator-led, open-label, single-arm clinical trial of adalimumab in moderate-to-severe HS (Hurley 2 and 3) (21). Skin biopsies were taken from lesional skin, defined as skin within 2 cm of an inflammatory nodule or abscess (**Table 1**). Patients with PsO, AD, and HC were recruited as part of a noninterventional case–control study with longitudinal biological sampling (**Figure 1A**). Skin biopsies were collected from the edge of a plaque of PsO or from an active, inflamed lesion in AD, as defined by experienced dermatologists, and from the arm, leg, or trunk of HC (**Table 2**). Samples were snap-frozen in liquid nitrogen and stored at − 80°C until RNA isolation was completed as previously described (9). Briefly, samples were pulverized using a BioPulveriser and homogenized in lysis buffer using a Precellys homogenizer. RNA was isolated using the Fibrous RNA Isolation Kit (Qiagen, The Netherlands) following the manufacturer’s instructions. High-quality samples (with an RNA integrity score of ≥ 6) were sent for bulk RNA-seq (Illumina HiSeq 4000; > 50 million 2 × 75 bp reads per sample) to AbbVie in Chicago, IL, USA (GSE306437). Analysis of raw RNA-sequencing data was completed using R Statistical Software (v4.2.1) (22) and Bioconductor packages (www.bioconductor.org). Data processing and normalization were performed using DESeq2. Batch effects were accounted for in the design of the DESeq2 analysis. Genes are considered significant with an adjusted *p*-value < 0.05 following Benjamini–Hochberg (BH) adjustment. EnrichR and pathfindR were used for pathway analysis, and the Weighted Gene Coexpression Network Analysis (WGCNA) package was used for gene coexpression analysis (23–27). Enriched pathways are considered significant if they have an adjusted *p*-value < 0.05 following BH adjustment. Cell type deconvolution was performed using xCell, which provides a prediction of cell type enrichment within a complex dataset (28). GraphPad Prism was used for the analysis of xCell scores and normalized count data. Analysis was completed using the Mann–Whitney statistical test for nonparametric unpaired data. A *p* ≤ 0.05 was considered significant. HS severity was defined by the International HS Severity Score (IHS4) classification (mild/moderate [*n* = 8], severe [*n* = 7]).

**Table 1:** Demographics and clinical details of HS, PsO, AD, and HC included in the RNA-seq analysis.

	HS (*n* = 15)	PsO (*n* = 21)	AD (*n* = 15)	HC (*n* = 19)	*p*-value
Female (*n*, %)	14 (93.3%)	5 (23.8%)	5 (33.3%)	11 (59%)	< 0.001^*^
Age (years; mean ± SD)	37.4 ± 9.7	47.9 ± 15.1	49.4 ± 17.1	32.8 ± 9.7	0.0007^*^
Smoking (*n*, %)					
Current	9 (60%)	4 (19%)	1 (6.7%)	4 (21.1%)	0.004^*^
Ex	4 (26.7%)	9 (42.9%)	8 (53.3%)	3 (15.8%)	
Non	2 (13.3%)	8 (38.1%)	5 (33.3%)	12 (63.2%)	
			1 (data not collected)		
Hurley stage (*n*, %)					
2	10 (66.6%)	n/a	n/a	n/a	n/a
3	5 (33.3%)				
PASI (mean ± SD)	n/a	11.8 ± 4.8	n/a	n/a	n/a
EASI (mean ± SD)	n/a	n/a	15.6 ± 8.2	n/a	n/a
BMI (kg/m^2^; mean ± SD)	35.1 ± 9.0 (*n* = 15)	27.8 ± 5.2 (*n* = 21)	26.5 ± 4.7 (*n* = 13)	25.3 ± 4.2 (*n* = 18)	< 0.0001^*^
			Data not collected (*n* = 2)	Data not collected (*n* = 1)	
Waist circumference (cm) (mean ± SD)	104.4 ± 26.8 (*n* = 15)	98.4 ± 13.5 (*n* = 21)	94.7 ± 14.7 (*n* = 13)	85 ± 12.1 (*n* = 18)	0.015^*^

Statistical significance was calculated using Pearson’s Chi-square for categorical data and one-way ANOVA for numerical data.

^*^ Significant *p*-values.

**Table 2:** Anatomical location of skin biopsy analyzed using bulk RNA-seq for baseline lesional HS, PsO, AD, and HC.

Biopsy location	HS (*n* = 15)	PsO (*n* = 21)	AD (*n* = 15)^a^	HC (*n* = 19)
Abdomen	1 (6.7%)	0	0	0
Arm	0	3 (14.3%)	5 (33.3%)	15 (78.9%)
Axilla	5 (33.3%)	0	0	0
Groin	4 (26.7%)	0	0	0
Intermammary	2 (13.3%)	0	0	0
Leg	0	12 (57.1%)	8 (53.3%)	3 (15.8%)
Thigh	3 (20%)	0	0	0
Trunk	0	6 (28.6%)	1 (6.7%)	1 (5.3%)

^a^ One biopsy location is not available.

Ethics approval was granted by the St. Vincent’s Healthcare Group Ethics and Medical Research Committee, and informed written consent was obtained from all participants prior to participation. Regulatory approval for the clinical trial (DERMMARK) was granted by the HPRA in Ireland. The trial was registered with the EU Clinical Trials Register (EudraCT Number 2016-001566028).

The data that support the findings of this study are openly available from an online data repository (GSE306437).

## Results

### Proinflammatory mediators predominate the transcriptomic differences in HS compared with HC skin

Principal component analysis (PCA) indicated that broad transcriptomic differences exist between HC (blue) and HS (mild/moderate, red; severe, dark red), PsO (orange), and AD (purple) skin, with severe HS displaying greater transcriptional heterogeneity than mild/moderate HS (**Figure 1B**). There were 4,937 differentially expressed genes (DEGs) (adjusted *p* ≤ 0.05) between HS and HC skin. A total of 2,641 genes were upregulated in HS, including antimicrobial peptides (S100 calcium-binding protein A (*S100A8*), *S100A9*), chemokines involved in neutrophil, T- and B-cell recruitment (C-X-C motif chemokine ligand (*CXCL1*), CC Chemokine Ligand (*CCL20*), *CXCL13*), and inflammatory cytokines and cytokine receptors (*TGFB1*, *IL-17F*, *IL-6*, *IL-36G*, *IL-23R*, *IL-1R1*) (**Figure 1C**). Gene ontology terms enriched from these DEGs include immunoglobulin production, B-cell-mediated immunity, and lymphocyte-mediated immunity (**Figure 1D**). Severe HS (IHS4 classification) had notably elevated expression of genes involved in immunoglobulin production relative to HC and mild/moderate HS, indicative of a prominent B- and plasma cell signature in severe HS (**Figure 1E**).

There were 801 DEGs shared between severe and mild/moderate HS relative to HC, 3,998 genes that were uniquely dysregulated in severe HS, and 554 genes that were dysregulated in mild/moderate HS relative to HC (**Figure 1F**). Dysregulated genes shared between severe and mild/moderate HS included *S100A8*, *S100A9*, *CCL20*, *CXCL1*, *IL-23R*, and Fibroblast Activation Protein (*FAP*) (**Figure 1G**). Th17-associated *IL-6* and *IL-36G* were uniquely dysregulated in mild/moderate HS relative to HC (**Figure 1G**). Elevated expression of *ICOS*, *CD69*, *IL-17F*, and *IFNG* indicates a prominent T-cell response in severe HS (**Figure 1G**). Additionally, increased Immunoglobulin Heavy Constant Gamma (*IGHG1*), Marginal Zone B and B1 Cell Specific Protein (*MZB1*), Joining Chain of Multimeric IgA and IgM (*JCHAIN*), *CXCL13*, and Bruton’s tyrosine kinase (*BTK*) expression was indicative of a prominent B-cell response in severe HS. Immune cell migration was enriched in both severe and mild/moderate HS, whereas B-cell-mediated immunity and immunoglobulin production were uniquely dysregulated in severe HS (**Figures 1H–J**).

### IL-17-mediated inflammation drives PsO; HS immunology is more heterogeneous

**Figure 2:**
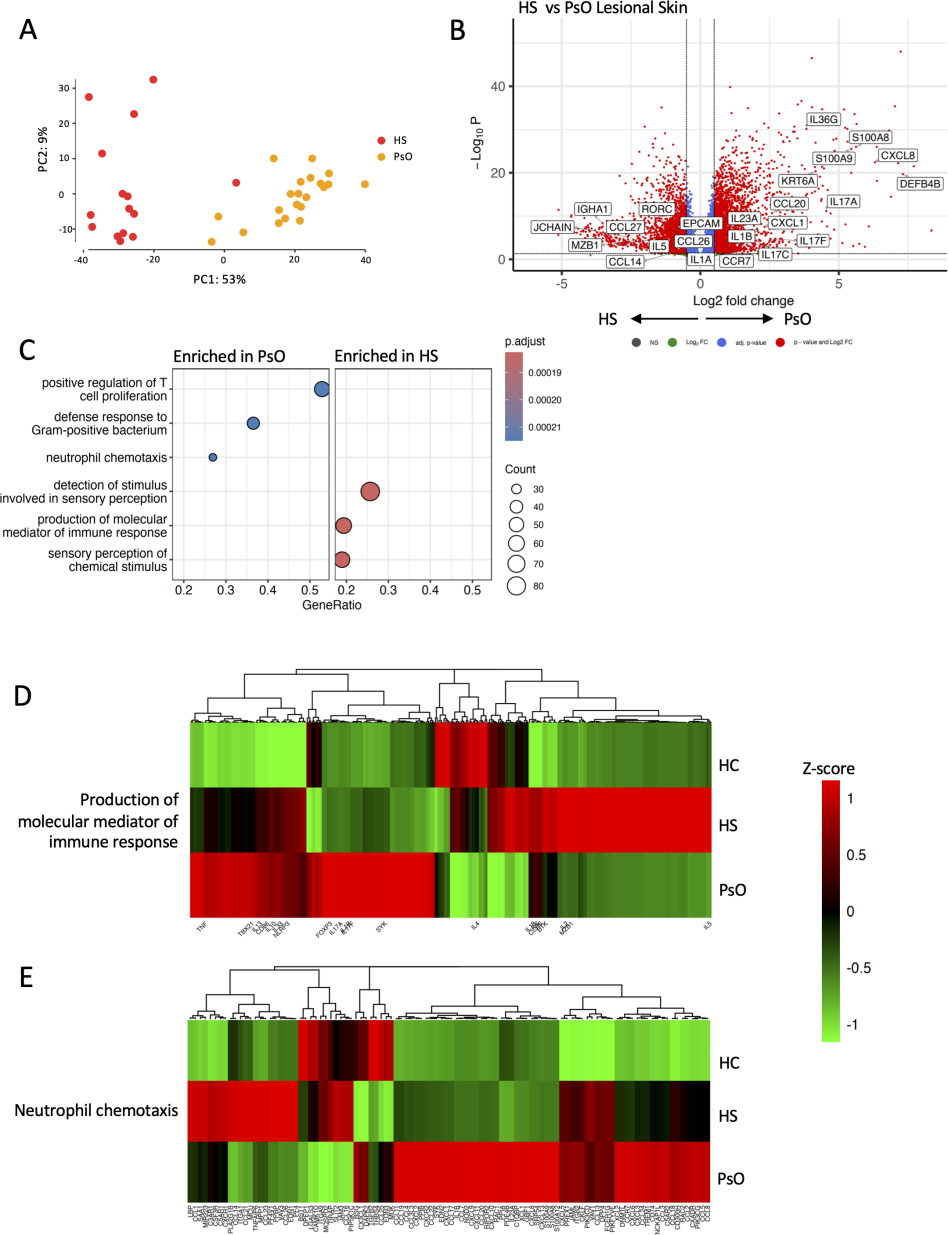
HS and psoriasis lesions have distinct transcriptomic profiles. Bulk RNA-seq was performed on HS (*n* = 15) and psoriasis lesions (*n* = 21; PsO). Principal component analysis demonstrates distinct transcriptomic profiles between HS and psoriasis lesions **(A)**. Differentially expressed genes between HS and psoriasis lesions visualized by a volcano plot. Genes with elevated expression in HS lesions have a negative log2 fold change, whereas genes with higher expression in psoriasis lesions have a positive fold change. The –log10 *p*-value on the *y*-axis represents the measure of significance **(B)**. A dotplot shows the top three gene ontology pathways enriched (*p*-value < 0.01 and *q*-value < 0.01 following BH adjustment) from the differentially expressed genes between HS and psoriasis lesions **(C)**. Heatmap displaying the relative expression of genes involved in the production of molecular mediators of immune response **(D)** and neutrophil chemotaxis pathways in HC, HS, and psoriasis lesions **(E)**.

PsO and HS were transcriptionally distinct (**Figure 2A**), emphasized by the 12,668 genes differentially expressed between HS and PsO. A total of 5,914 genes were upregulated in PsO, and 6,754 genes were upregulated in HS. IL-17-associated genes (*IL-17A*, *IL-23A*, *S100A8*, Keratin 6A (*KRT6A*), *CXCL8*, *CCL20*) and *IL-1B* were elevated in PsO (**Figure 2B**). Conversely, HS exhibited an upregulation of B- and plasma cell-associated genes (*JCHAIN*, *MZB1*), Nucleotide-binding oligomerization domain, Leucine-rich Repeat and Pyrin domain containing (*NLRP1*), and IL-17-associated *IL-17RC* and *RORC* (**Figure 2B**). T-cell proliferation and neutrophil chemotaxis were enriched in PsO, whereas the production of molecular mediators of immune response was most significantly enriched in HS (**Figures 2C–E**). Pronounced transcriptomic differences were identified between HS severities and PsO (**Supplementary Figures 1A, B**). Notably, mild/moderate HS had increased expression of the skin-homing chemokine receptor *CCR10* and the IL-17A receptor *IL-17RC*, while severe HS had elevated expression of *BTK*, *CD79B*, and *C3* (**Supplementary Figure 1C**). Importantly, B- and plasma cell-associated pathways were enriched in severe HS relative to PsO (**Supplementary Figure 1E**), while mild/moderate HS had an enrichment of NK cell-mediated pathways when compared with PsO (**Supplementary Figure 1F**).

**Figure 3:**
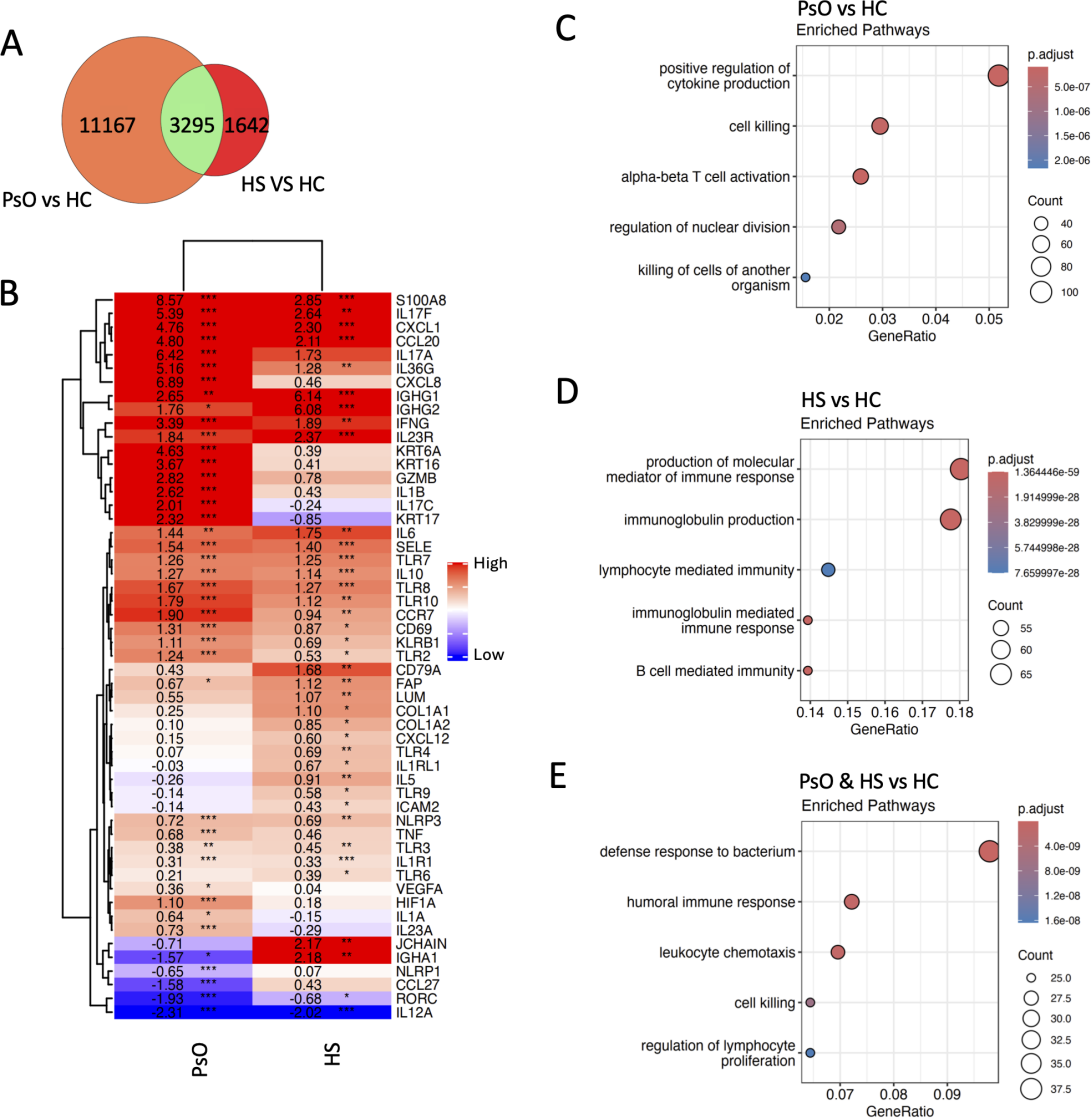
B-cell signaling differentiates HS inflammation from psoriasis. Bulk RNA-seq was performed on HS (*n* = 15) and psoriasis lesions (*n* = 21). Venn diagram illustrating the shared differentially expressed genes between HS and psoriasis lesions when compared to HC skin **(A)**. Heatmap displaying the log2 fold changes of inflammatory genes in HS and psoriasis lesions relative to HC skin. Statistical significance was calculated by differential gene expression in DeSeq2 **(B)**. ^*^p ≤ 0.05; ^**^p ≤ 0.01; ^***^p ≤ 0.001. Top five gene ontology terms enriched from the differentially expressed genes (both upregulated and downregulated) unique to psoriasis **(C)** and HS **(D)** or shared between them when compared with HC skin (*p*-value < 0.01 and *q*-value < 0.01 following BH adjustment) **(E)**.

To reveal differences between HS and PsO pathogenesis, HS and PsO were individually compared with HC skin. There were 3,295 genes shared between HS and PsO, 11,167 genes that were only differentially expressed in PsO, including *IL-17A*, *IL-17C*, *IL-1B*, and *IL-23A*, and 1,642 genes that were only differentially expressed in HS, including most immunoglobulin genes, *CD79A* and *JCHAIN* (**Figure 3A**). Dysregulated genes shared in both HS and PsO pathogenesis included IL-17 signaling genes (*IL-17F*, *CCL20*, *IL-23R*, *IL-12A*, *KLRB1*, *CCR7*, *CXCL1*, *S100A8*), complement genes, *NLRP3*, *IFNG*, and *IL-36G* (**Figure 3B**). Gene ontology terms enriched in PsO were related to T-cell activation and cytokine production (**Figure 3C**), while B-cell-mediated immunity and immunoglobulin production were enriched in HS (**Figure 3D**). Humoral immune response and immune cell migration pathways were enriched in genes dysregulated in both HS and PsO (**Figure 3E**).

### B-cell and TNF signatures differentiate HS from atopic dermatitis

**Figure 4:**
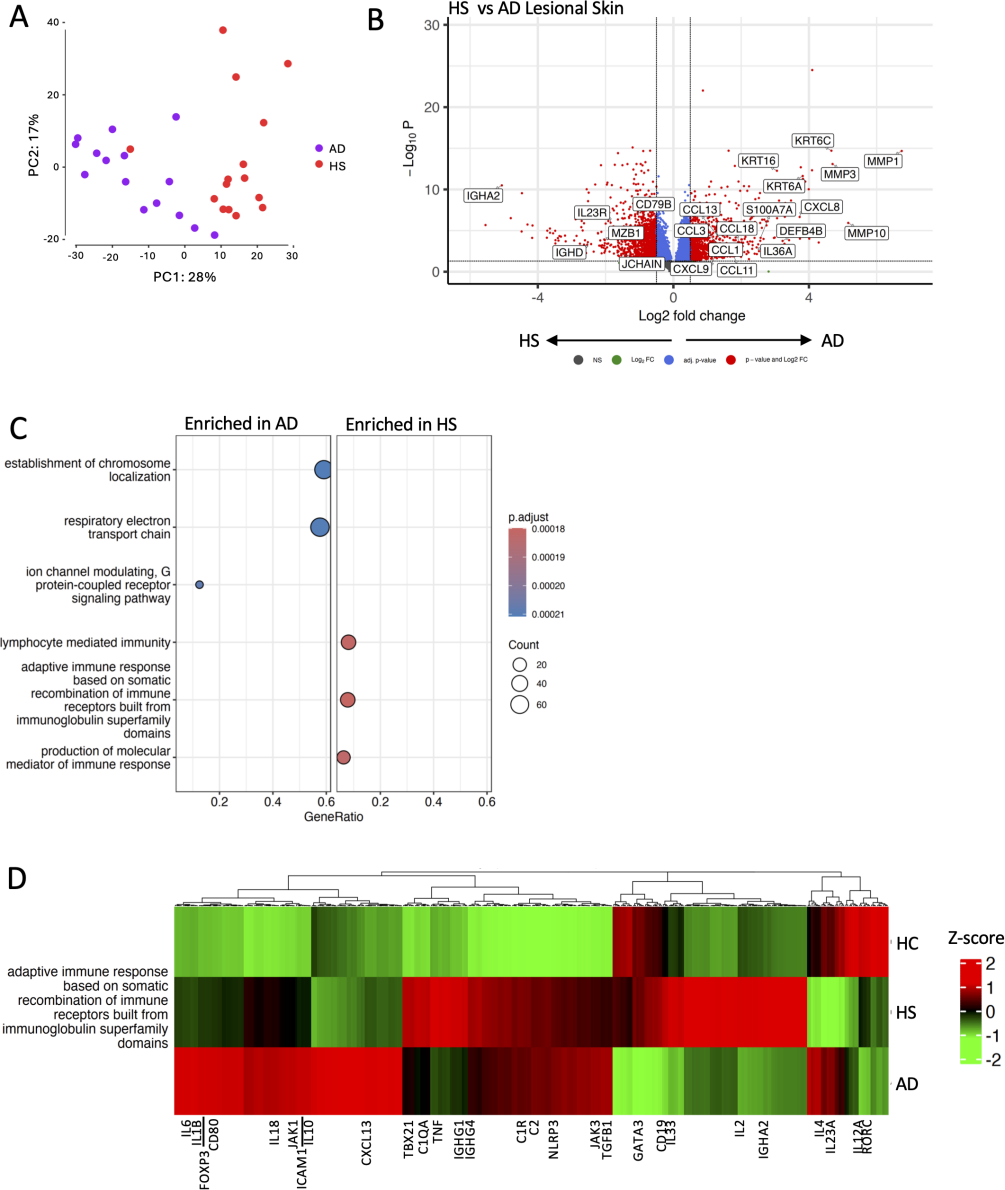
HS and atopic dermatitis lesions have a distinct transcriptomic profile. Bulk RNA-seq was performed on HS (*n* = 15) and psoriasis lesions (*n* = 15; AD). Principal component analysis demonstrates distinct transcriptomic profiles between HS and atopic dermatitis lesions **(A)**. Differentially expressed genes between HS and atopic dermatitis lesions are visualized by a volcano plot. Genes with elevated expression in HS lesions have a negative log2 fold change, whereas genes with higher expression in atopic dermatitis lesions have a positive fold change. The –log10 *p*-value on the *y*-axis represents the measure of significance **(B)**. Dotplot displaying the top three gene ontology pathways enriched (*p*-value < 0.01 and *q*-value < 0.01 following BH adjustment) from the differentially expressed genes between HS and atopic dermatitis lesions **(C)**. Heatmap displaying the relative expression of genes involved in the adaptive immune response based on somatic recombination of immune receptors built from immunoglobulin superfamily domains pathway **(D)**.

PCA demonstrated overall transcriptomic differences between HS and AD (**Figure 4A**). A total of 7,192 genes were differentially expressed between AD and HS. AD had 3,709 genes upregulated, including Th2-associated *IL-4R*, *IL-13*, and *IL-13RA1*, matrix metalloproteinases (*MMP1*, *MMP3*, *MMP10)*, *CXCL8*, *KRT6A*, and antimicrobial peptides (*S100A8* and *S100A9*) (**Figure 4B**). HS had 3,483 genes upregulated, including inflammasome-associated *NLRP1* and *GSDMD*, *IL-33*, and *MZB1*, a B-cell-associated gene (**Figure 4B**). The electron transport chain was enriched in AD, whereas lymphocyte-mediated immunity and immunoglobulin-associated pathways were enriched in HS (**Figure 4C**). Genes involved in immunoglobulin production were highly dysregulated in HS relative to HC and AD (**Figure 4D**). Interestingly, mild/moderate HS had increased expression of GATA Binding Protein 3 (*GATA3*) relative to both severe HS and AD, indicating a skewed Th2 profile (**Supplementary Figure 2C**), while *BTK* expression and B- and plasma cell pathways were notably enriched in severe HS, indicating a potential therapeutic angle for individuals with severe HS (**Supplementary Figures 2C, E**).

**Figure 5:**
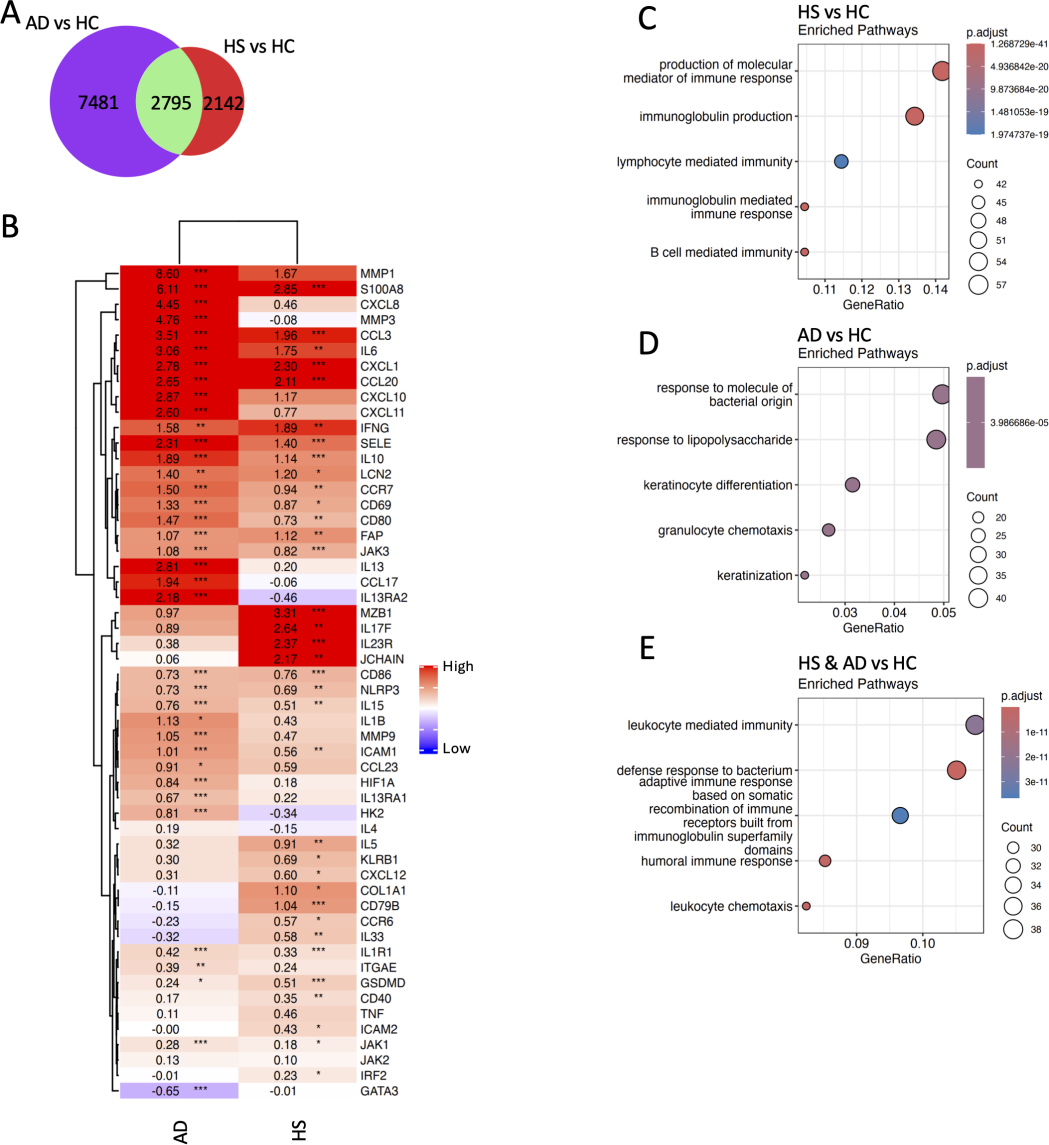
HS and AD lesions have distinct drivers of inflammation. Bulk RNA-seq was performed on HS (*n* = 15) and AD lesions (*n* = 15). Venn diagram illustrating the shared differentially expressed genes between HS and AD lesions when compared to HC skin **(A)**. Heatmap displaying the log2 fold changes of inflammatory genes in HS and AD lesions relative to HC skin. Statistical significance was calculated by differential gene expression in DeSeq2 **(B)**. ^*^p ≤ 0.05; ^**^p ≤ 0.01; ^***^p ≤ 0.001. Top five gene ontology terms enriched from the differentially expressed genes (both up- and downregulated) unique to AD **(C)** and HS **(D)** or shared between them when compared with HC skin (*p*-value < 0.01 and *q*-value < 0.01 following BH adjustment) **(E)**.

To identify differences between HS and AD pathogenesis, HS and AD skin were compared with HC skin. A total of 7,481 genes were uniquely differentially expressed in AD in comparison with HC skin. AD showed increased expression of Th2-associated genes (*IL-13*, *GATA3*, *IL-13RA1*, *IL-13RA2*), *IL-1B*, *CXCL8*, *CXCL11*, and *CCL17*, a T-cell chemoattractant (**Figure 5A**). HS and AD shared 2,795 DEGs when compared with HC skin, including inflammatory mediators *IL-6*, *IFNG*, and *NLRP3*; IL-17-associated genes *CCL20*, *CXCL1*, and *S100A8;* and Janus kinase (JAK) signaling genes (*JAK1*, *JAK3*) (**Figure 5B**). There were 2,142 genes uniquely dysregulated in HS, including *IL-17F*, *IL-23R*, *KLRB1* (CD161), *COL1A1*, and *CCL5*, a monocyte and effector memory T-cell chemoattractant (29) (**Figure 5B**). B- and plasma cell-associated genes *JCHAIN*, *CD79B*, and immunoglobulins were upregulated in HS skin compared with AD. These findings were confirmed with an enrichment of B-cell-mediated immunity in HS and leukocyte-mediated immunity enriched in both HS and AD (**Figures 5C–E**).

### B- and plasma cell signatures distinguish HS from other inflammatory skin diseases

PsO and AD have distinct transcriptomic profiles primarily driven by alternative T-cell signaling (**Supplementary Figures 3A, B**). PsO has increased expression of IL-17-associated genes (*IL-17A*, *IL-17F*, *IL-17C*, *IL-23R*, *KLRB1*, *CXCL8*, *S100A8*, *S100A9*, *CCL20*), likely promoted by a heightened innate immune response (**Supplementary Figure 3C**). Conversely, AD has elevated expression of the Th2 cytokine *IL-13* and MMPs, indicating fibroblasts may be dysregulated in AD (**Supplementary Figure 3B**). AD and PsO share 8,389 DEGs when compared with HC, including MMPs, *FAP*, *ICAM1*, and S100s, suggesting shared stromal cell dysregulation in both AD and PsO (**Supplementary Figure 3D**). Of note, *IL-17A*, *IL-17F*, *IL-23A*, *IL-1A*, and *IL-33* were uniquely upregulated in PsO, reaffirming the prominent role of IL-17 and IL-1 signaling in PsO. Pathway analysis demonstrated that T-cell activation was enriched in PsO, whereas AD showed an enrichment of cell chemotaxis pathways, indicative of immune cell infiltration in AD (**Supplementary Figures 3F, G**). Pathways enriched from the DEGs shared between PsO and AD were associated with a humoral immune response and response to LPS, demonstrating commonalities between these dermatoses (**Supplementary Figure 3H**).

**Figure 6:**
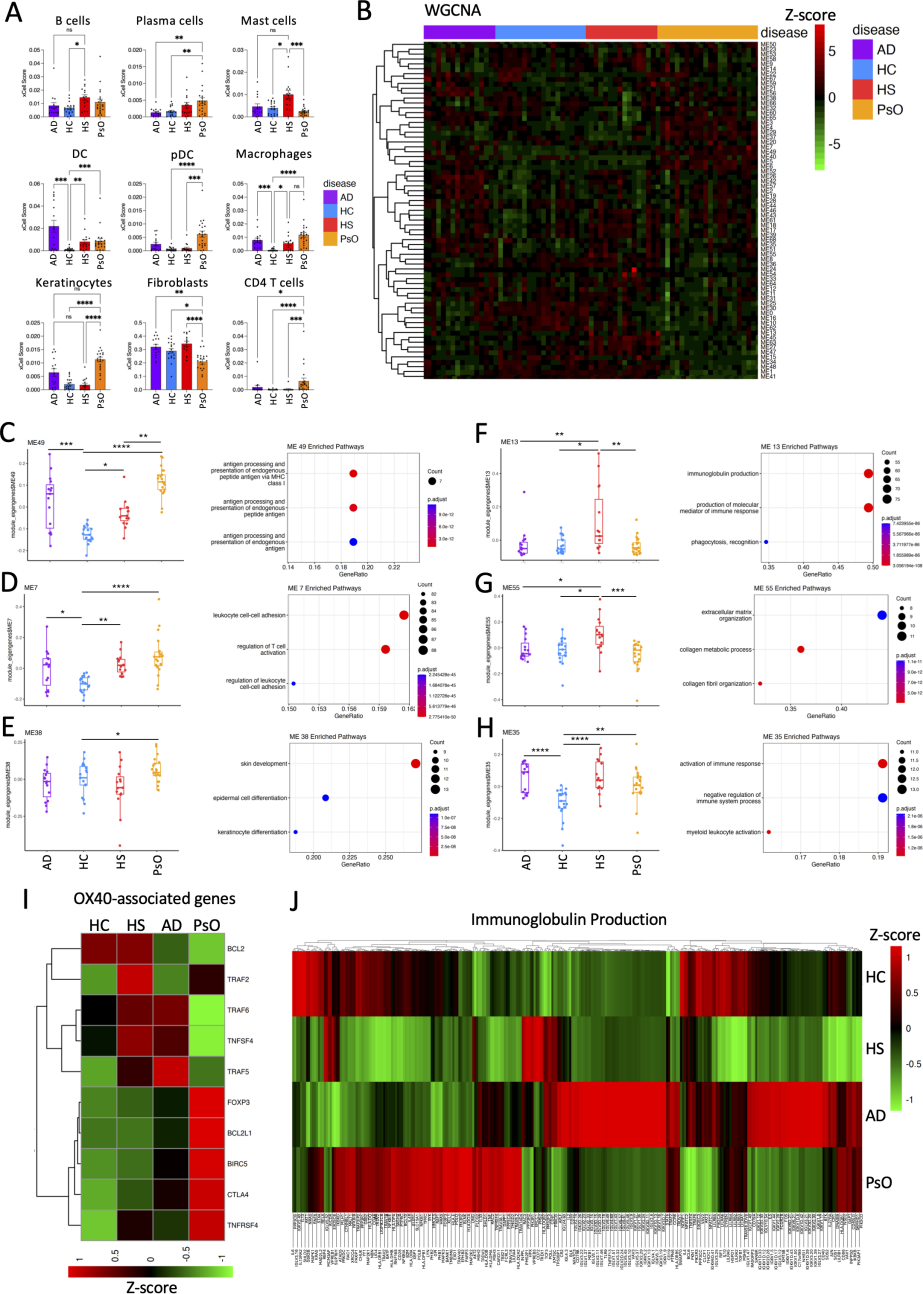
B- and plasma cell signature distinguish HS from other inflammatory dermatoses. Bulk RNA-seq was performed on HC skin (*n* = 19), HS (*n* = 15), psoriasis (*n* = 21; PsO), and AD lesions (*n* = 15). Cell type deconvolution using xCell predicts the enrichment of cell populations from bulk RNA-seq data. Graphs represent individual samples with mean ± SEM for each group. Statistical significance was calculated using Kruskal–Wallis one-way ANOVA with Dunn’s multiple comparisons test **(A)**. ^*^p ≤ 0.05; ^**^p ≤ 0.01; ^***^p ≤ 0.001; ^****^p ≤ 0.0001. Weighted gene coexpression analysis (WGCNA) of bulk RNA-seq data **(B)**. Boxplot of gene modules significantly dysregulated between HC, AD, HS, and PsO samples, along with the top three gene ontology terms enriched from genes in the significantly dysregulated gene modules (*p*-value < 0.01 and *q*-value < 0.01 following BH adjustment) **(C–H)**. Heatmaps displaying the relative expression of genes involved in the OX-40 pathway **(I)** and OX-40 pathway immunoglobulin production **(J)** in HC, HS, PsO, and AD.

AD had a significant enrichment of DCs and macrophages, while plasma cells, DCs, plasmacytoid DCs, macrophages, keratinocytes, and CD4 T cells were enriched in PsO relative to HC (**Figure 6A**). HS had an enrichment of B cells, mast cells, DCs, and macrophages (**Figure 6A**), consistent with findings from Lowe et al. (30). WGCNA generates modules of strongly correlated genes (**Figure 6B**) (27). Gene modules prominent in AD were associated with antigen presentation, T-cell activation, and myeloid cell activation (**Figure 6C**). PsO also had a gene module related to epidermal development that was significantly upregulated relative to HC (**Figure 6E**). Highlighting the heterogeneity of HS immunopathogenesis, HS showed an enrichment of antigen presentation (**Figure 6C**), T-cell activation (**Figure 6D**), immunoglobulin production (**Figure 6F**), extracellular matrix organization (**Figure 6G**), and myeloid cell activation (**Figure 6H**). Each inflammatory dermatosis demonstrated dysregulated expression of genes involved in OX-40 signaling, potentially illustrating a mechanistic link among these three distinct inflammatory skin diseases. A B- and plasma cell signature distinguishes HS from the other chronic inflammatory skin disorders (**Figure 6J**).

## Discussion

Single cytokine inhibition therapies have been highly effective in PsO and AD; however, more modest efficacy has been observed in HS, and a large unmet therapeutic need still exists.

In this study, we compared the transcriptomic signature in HS, AD, and PsO, and, consistent with other studies, we identified an enrichment of B-cell-associated genes in HS (31, 32). Differential gene expression analysis outlined pathways with therapeutic potential in HS, including IFN-γ, IL-36γ, B cell, Th17, IL-6, and neutrophil signaling.

The increased expression of *IL-17A*, *IL-23A*, *CCL20*, and *CXCL8* indicates why targeting IL-17 signaling has proven to be more successful than anti-TNF treatment in PsO, while the expression of B-cell-, inflammasome-, and T-cell-associated genes in HS suggests a more heterogeneous immunological landscape exists in HS. Both HS and PsO exhibit increased T-cell and humoral immune response, including *CD69*, *KLRB1*, *IL-17F*, *IFNG*, *IGHG1*, *IGHG2*, and *IGHA1*, providing potential insight into how to effectively target the minority of patients who have concomitant PsO and HS (33). A strong B- and plasma cell signature identifies a cohort of patients with severe HS who may benefit from B-cell depletion or BTK and SYK inhibitors (31). This is supported by previous studies demonstrating an influx of B cells in HS-inflamed skin and the therapeutic potential of B-cell depletion in HS (34, 35). Previous findings have highlighted an upregulation of complement and B-cell-associated genes in patients who failed to respond to adalimumab treatment, enforcing the importance of patient stratification (21).

AD was defined by a Th2 signature and increased expression of *CXCL8*, *KRT6A*, and *S100A8*, while HS had increased *IL*-23, IL-1, and B-cell signaling. IL-17F, which has become a therapeutic target of interest in HS (15), was upregulated in HS, as were immunoglobulin and B-cell-associated genes, highlighting a potentially important role of Th17 and B cells in HS inflammation. Notably, JAK1 and JAK3 were elevated in both HS and AD, supporting the recent positive results of povorcitinib and upadacitinib in HS and the approval of JAK-1 inhibitors in AD.

T-cell signaling and epidermal development were enriched in PsO, coinciding with the dramatic epidermal thickening seen in PsO. AD had elevated Th2 cells, while HS had an enrichment of B cells, mast cells, fibroblasts, and macrophages. Our data highlight a B- and plasma cell signature that is unique to HS and is not found in PsO or AD. Consistent with the heterogeneity seen among patients with HS, this B- and plasma cell signature is more enhanced in a cohort of patients with HS, indicating the potential to stratify patients based on the presence of this B- and plasma cell signature.

Our study reports the novel findings of the upregulation of JAK-1 and JAK-3 pathways and OX-40L in HS. This supports the rationale for the ongoing clinical trials of agents that target these pathways in HS.

Our study was limited by the sample size and the lack of matching body mass index (BMI) and smoking across disease groups. Our findings are broadly supported by other studies, which highlight the role of B and plasma cells in HS (31, 36, 37). Smoking, BMI, sex, and anatomical site of biopsy are potential confounding factors that are known to impact inflammatory pathways and gene expression profiles and may contribute to the transcriptomic differences described here. While this study demonstrates a clear signature that is unique to HS, single-cell and spatial transcriptomics would confirm that this signature results from the transcriptional activation of specific B- and plasma cell subsets.

This study illustrates the heterogeneity of HS pathogenesis and the importance of patient stratification to identify patients who will optimally respond to targeted treatments. While targeting Th2 cytokines in AD and IL-17 signaling in PsO has proven to be an effective strategy, a larger arsenal of therapeutics, together with patient stratification, may be required to combat the disease heterogeneity that exists in HS. The increasing appreciation of the role of B and plasma cells in HS inflammation generated by this study and others provides support for the potential use of B-cell signaling inhibitors or B-cell depletion for the treatment of HS.

## Data Availability

The datasets presented in this study can be found in online repositories. The names of the repository/repositories and accession number(s) can be found below: GSE306437 (GEO).

## Supplementary material

Supplementary Figure 1 B and plasma cell pathways dominate severe HS. Bulk RNA-seq was performed on HS (n=15) and PsO lesions (n=21). Bar chart displaying the number of DEGs elevated in HS (red) or PsO (orange) when comparing mild/moderate and severe HS to PsO **(A)**. Venn diagram illustrating the shared differentially expressed genes between HS and PsO lesions when compared to HC skin **(B)**. Heatmap displaying the log2 fold changes of inflammatory genes in mild/moderate and severe HS relative to PsO lesions. Statistical significance was calculated by differential gene expression in DeSeq2 **(C)**; * p≤ 0.05, ** p≤ 0.01, *** p≤ 0.001, **** p≤ 0.0001. Top 5 gene ontology terms enriched from the differentially expressed genes unique to mild/moderate **(F)** and severe HS **(E)** or shared between them when compared with PsO (p value<0.01 & q value <0.01 following BH adjustment) **(D)**.

Supplementary Figure 2 Keratinocyte dysregulation is a distinguishing feature of HS compared to AD, regardless of HS severity. Bulk RNA-seq was performed on HS (n=15) and AD lesions (n=15). Bar chart displaying the number of DEGs elevated in HS (red) or AD (purple) when comparing mild/moderate and severe HS to AD. Venn diagram illustrating the shared differentially expressed genes between HS and AD lesions when compared to HC skin **(B)**. Heatmap displaying the log2 fold changes of inflammatory genes in mild/moderate and severe HS relative to AD lesions. Statistical significance was calculated by differential gene expression in DeSeq2 **(C)**; * p≤ 0.05, ** p≤ 0.01, *** p≤ 0.001, **** p≤ 0.0001. Top 5 gene ontology terms enriched from the differentially expressed genes unique to mild/moderate **(F)** and severe HS **(E)** or shared between them when compared with AD (p value<0.01 & q value <0.01 following BH adjustment) **(D)**.

Supplementary Figure 3 Distinct T cell pathways drive AD and PsO. Bulk RNA-seq was performed on PsO (n=19) and AD lesions (n=15). Principal component analysis demonstrates distinct transcriptomic profiles between PsO and AD lesions **(A)**. Differentially expressed genes between PsO and AD lesions visualized by volcano plot. Genes with elevated expression in PsO lesions have a negative Log2 fold change, genes with higher expression in atopic dermatitis lesions have a positive fold change. The –Log10 P Y axis is a measure of significance **(B)**. Dotplot displaying top 3 gene ontology pathways enriched (p value<0.01 & q value <0.01 following BH adjustment) from the differentially expressed genes between HS and atopic dermatitis lesions **(C)**. Venn diagram illustrating the shared differentially expressed genes between PsO and AD lesions when compared to HC skin **(D)**. Heatmap displaying the log2 fold changes of inflammatory genes in PsO and AD lesions relative to HC skin **(E)**. Statistical significance was calculated by differential gene expression in DeSeq2 **(B)**; * p≤ 0.05, ** p≤ 0.01, *** p≤ 0.001, **** p≤ 0.0001. Top 5 gene ontology terms enriched (p value<0.01 & q value <0.01 following BH adjustment) from the differentially expressed genes unique to PsO **(F)** and AD **(G)** or shared between them when compared with HC skin **(H)**.
