# Supplementary figures and images for: Unique B- and plasma cell signature differentiates hidradenitis suppurativa from psoriasis and atopic dermatitis
Source: Front Immunol. 2026 Apr 16;17:1768249. doi: 10.3389/fimmu.2026.1768249 (PMC13128555; doi:10.3389/fimmu.2026.1768249)

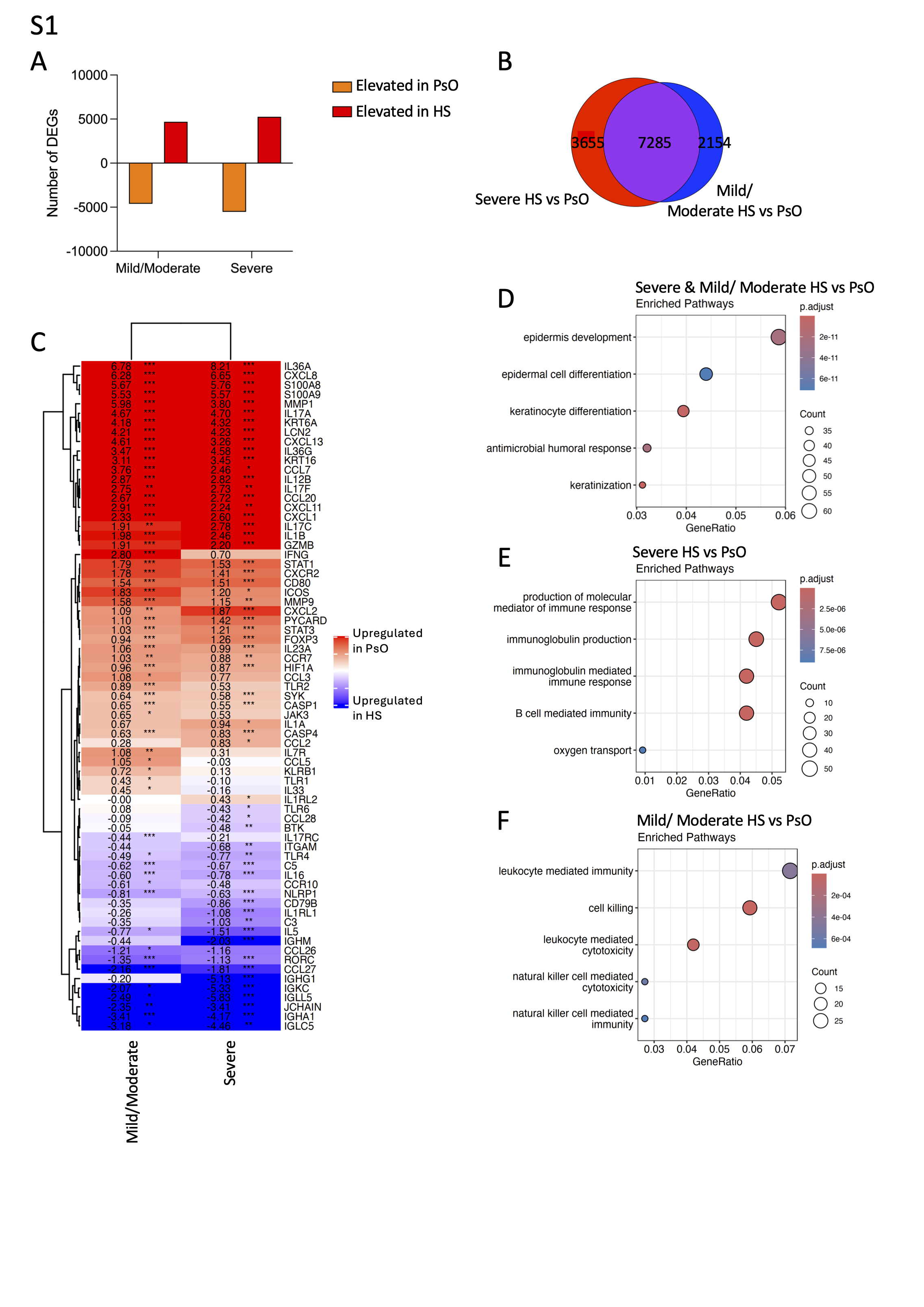

Supplement: Supplementary Figure 1 — B and plasma cell pathways dominate severe HS. Bulk RNA-seq was performed on HS (n=15) and PsO lesions (n=21). Bar chart displaying the number of DEGs elevated in HS (red) or PsO (orange) when comparing mild/moderate and severe HS to PsO (A). Venn diagram illustrating the shared differentially expressed genes between HS and PsO lesions when compared to HC skin (B). Heatmap displaying the log2 fold changes of inflammatory genes in mild/moderate and severe HS relative to PsO lesions. Statistical significance was calculated by differential gene expression in DeSeq2 (C); * p≤ 0.05, ** p≤ 0.01, *** p≤ 0.001, **** p≤ 0.0001. Top 5 gene ontology terms enriched from the differentially expressed genes unique to mild/moderate (F) and severe HS (E) or shared between them when compared with PsO (p value<0.01 & q value <0.01 following BH adjustment) (D). [file Image1.tiff]

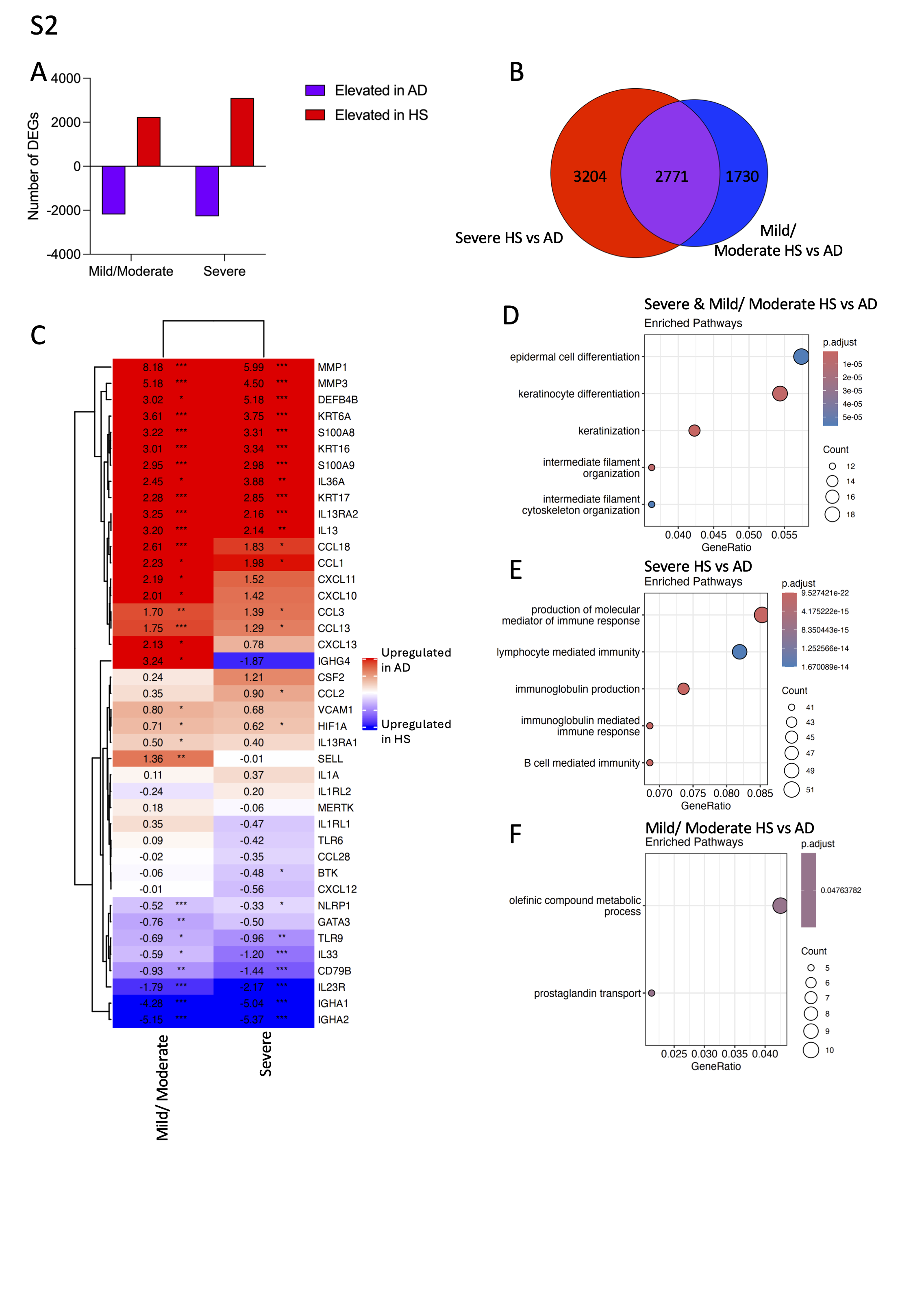

Supplement: Supplementary Figure 2 — Keratinocyte dysregulation is a distinguishing feature of HS compared to AD, regardless of HS severity. Bulk RNA-seq was performed on HS (n=15) and AD lesions (n=15). Bar chart displaying the number of DEGs elevated in HS (red) or AD (purple) when comparing mild/moderate and severe HS to AD. Venn diagram illustrating the shared differentially expressed genes between HS and AD lesions when compared to HC skin (B). Heatmap displaying the log2 fold changes of inflammatory genes in mild/moderate and severe HS relative to AD lesions. Statistical significance was calculated by differential gene expression in DeSeq2 (C); * p≤ 0.05, ** p≤ 0.01, *** p≤ 0.001, **** p≤ 0.0001. Top 5 gene ontology terms enriched from the differentially expressed genes unique to mild/moderate (F) and severe HS (E) or shared between them when compared with AD (p value<0.01 & q value <0.01 following BH adjustment) (D). [file Image2.tiff]

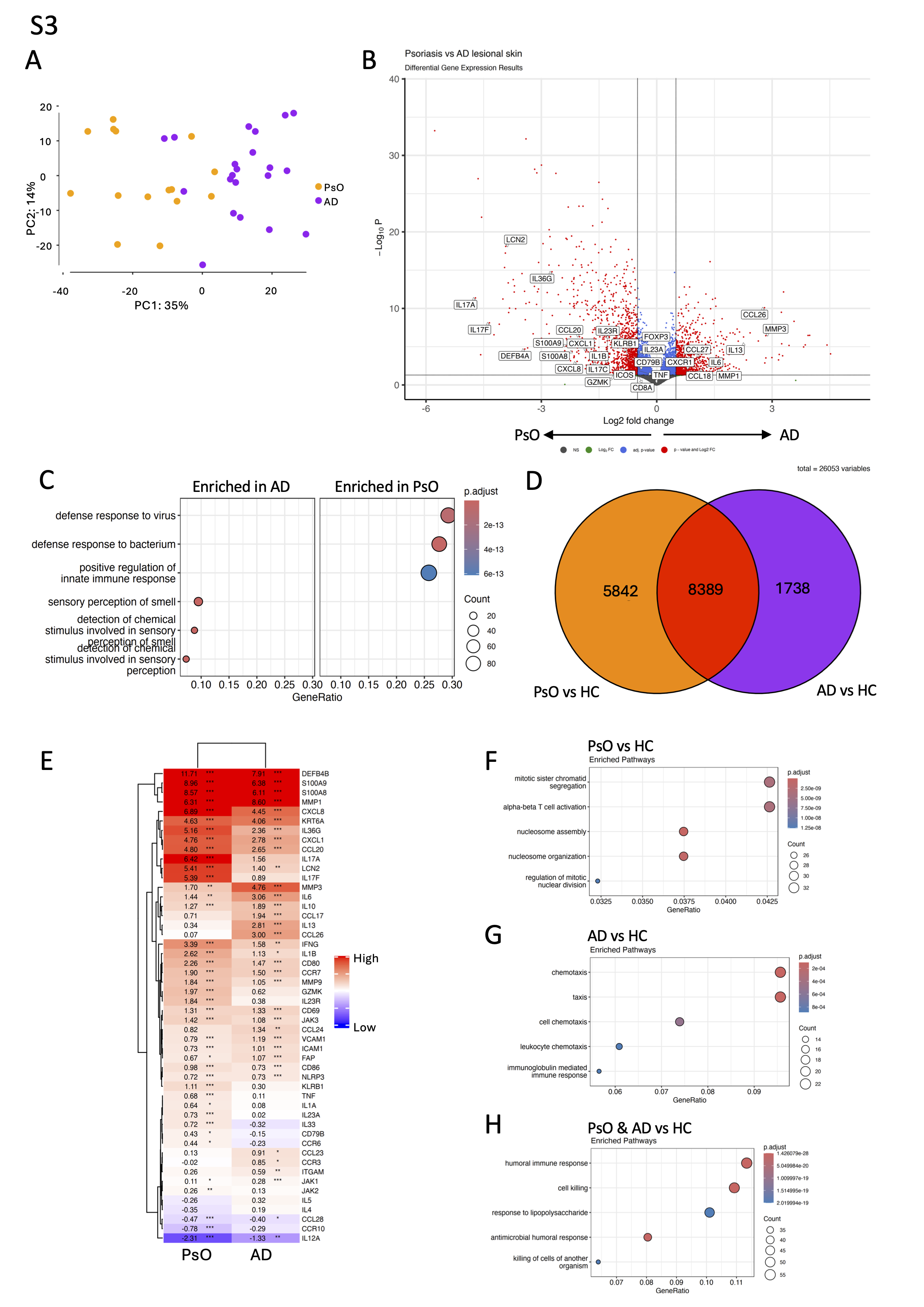

Supplement: Supplementary Figure 3 — Distinct T cell pathways drive AD and PsO. Bulk RNA-seq was performed on PsO (n=19) and AD lesions (n=15). Principal component analysis demonstrates distinct transcriptomic profiles between PsO and AD lesions (A). Differentially expressed genes between PsO and AD lesions visualized by volcano plot. Genes with elevated expression in PsO lesions have a negative Log2 fold change, genes with higher expression in atopic dermatitis lesions have a positive fold change. The –Log10 P Y axis is a measure of significance (B). Dotplot displaying top 3 gene ontology pathways enriched (p value<0.01 & q value <0.01 following BH adjustment) from the differentially expressed genes between HS and atopic dermatitis lesions (C). Venn diagram illustrating the shared differentially expressed genes between PsO and AD lesions when compared to HC skin (D). Heatmap displaying the log2 fold changes of inflammatory genes in PsO and AD lesions relative to HC skin (E). Statistical significance was calculated by differential gene expression in DeSeq2 (B); * p≤ 0.05, ** p≤ 0.01, *** p≤ 0.001, **** p≤ 0.0001. Top 5 gene ontology terms enriched (p value<0.01 & q value <0.01 following BH adjustment) from the differentially expressed genes unique to PsO (F) and AD (G) or shared between them when compared with HC skin (H). [file Image3.tiff]
